# Association between patient‐initiated emails and overall 2‐year survival in cancer patients undergoing chemotherapy: Evidence from the real‐world setting

**DOI:** 10.1002/cam4.3483

**Published:** 2020-09-28

**Authors:** Jean Coquet, Douglas W. Blayney, James D. Brooks, Tina Hernandez‐Boussard

**Affiliations:** ^1^ Department of Medicine Stanford University Stanford CA USA; ^2^ Stanford Cancer Institute Stanford University School of Medicine Stanford CA USA; ^3^ Department of Urology Stanford University School of Medicine Stanford CA USA; ^4^ Department of Biomedical Data Science Stanford University Stanford CA USA; ^5^ Department of Surgery Stanford University School of Medicine Stanford CA USA

**Keywords:** chemotherapy, communication, email, survival

## Abstract

**Purpose:**

Prior studies suggest email communication between patients and providers may improve patient engagement and health outcomes. The purpose of this study was to determine whether patient‐initiated emails are associated with overall survival benefits among cancer patients undergoing chemotherapy.

**Patients and methods:**

We identified patient‐initiated emails through the patient portal in electronic health records (EHR) among 9900 cancer patients receiving chemotherapy between 2013 and 2018. Email users were defined as patients who sent at least one email 12 months before to 2 months after chemotherapy started. A propensity score‐matched cohort analysis was carried out to reduce bias due to confounding (age, primary cancer type, gender, insurance payor, ethnicity, race, stage, income, Charlson score, county of residence). The cohort included 3223 email users and 3223 non‐email users. The primary outcome was overall 2‐year survival stratified by email use. Secondary outcomes included number of face‐to‐face visits, prescriptions, and telephone calls. The healthcare teams’ response to emails and other forms of communication was also investigated. Finally, a quality measure related to chemotherapy‐related inpatient and emergency department visits was evaluated.

**Results:**

Overall 2‐year survival was higher in patients who were email users, with an adjusted hazard ratio of 0.80 (95 CI 0.72–0.90; *p* < 0.001). Email users had higher rates of healthcare utilization, including face‐to‐face visits (63 vs. 50; *p* < 0.001), drug prescriptions (28 vs. 21; *p* < 0.001), and phone calls (18 vs. 16; *p* < 0.001). Clinical quality outcome measure of inpatient use was better among email users (*p* = 0.015).

**Conclusion:**

Patient‐initiated emails are associated with a survival benefit among cancer patients receiving chemotherapy and may be a proxy for patient engagement. As value‐based payment models emphasize incorporating the patients’ voice into their care, email communications could serve as a novel source of patient‐generated data.

## Graphical abstract

Patient portals provide a tool for patients to communicate with their care team that provides overall clinical benefit for advanced cancer patients; the healthcare team's response to patients’ emails included drug prescriptions, medical appointment scheduling, telephone calls, and response emails. Patient emails are a novel source of patient‐generated data that may play a vital role in patient symptom monitoring.

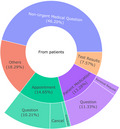

## 1 INTRODUCTION

Email use has transformed routine and transactional communication between patients and providers,^1^ supplanting telephone and paper‐based communication. As internet use is ubiquitous, fast and readily available, email communication is convenient for both the patient and provider because it is asynchronous (both parties do not have to be simultaneously available for effective communication) and because email can be spontaneous—the subject and content are determined by the sender.^2^ The use of email as a way to communicate is widely accepted by patients in their daily lives and by healthcare systems and practitioners for patient communication. As email becomes the standard for communication, its integration into the healthcare system will continue to expand.

With the ubiquitous expansion of the electronic health record (EHR) most healthcare delivery systems have adopted patient portals to improve patients’ access to healthcare information and promote active partnerships between patients and providers and patients have rapidly embraced this opportunity.^3^, ^4^ These systems provide secure, always‐on, private access to patient information. The EHR also facilitates convenient, secure, asynchronous intra‐health system communication among providers. Studies suggest that portal use may increase treatment adherence, reduce medical errors and adverse drug reactions, facilitate patient–provider communication, and increase patient engagement, perception of care quality, and autonomy—all associated with better outcomes.^5^, ^6^ Importantly, most patient portals are equipped with automated patient messaging systems, allowing patients to ask questions of their physicians and other healthcare providers.

Healthcare portals allow patients to send non‐urgent medical questions to their care team via an email‐like feature. These inbound, patient‐generated emails are a new form of information which allows patients to communicate—in their own words—with their healthcare team at a time convenient for the patient. Unlike physician or other provider generated narrative text, patient‐generated emails are in the patients’ voice without interpretation by medical staff. A recent study classified patient emails based on their content and found emails were commonly sent for medical follow‐up, appointment scheduling, and medication refills, but also included a large group of unclassified emails.^7^ Understanding how patient‐generated emails enhance engagement and whether they can affect patient outcomes is essential in a value‐based care setting, particularly for patients dealing with serious illnesses such as advanced cancer.

Our study evaluated this novel source of patient‐generated data: cancer chemotherapy patient‐generated emails to the healthcare team using the patient portal. We quantified email use among chemotherapy patients, characterized email types, assessed the healthcare teams’ response to patient‐generated emails, tested whether email usage was associated with interactions with the healthcare system, and evaluated potential survival benefits of patients who initiated emails.

## 2 METHODS

### 2.1 Data source

The data for this study were derived from the EHRs of patients receiving chemotherapy treatment at the Stanford Cancer Institute. Stanford has a fully implemented EPIC EHR system, installed in 2008.^8^ As part of the EHR system, the MyHealth portal and web interface was fully integrated with the EHR in 2012, including a patient portal that allows, inter alia, patients to communicate with their healthcare team via emails. This study received the approval from the institute's Institutional Review Board (IRB).

### 2.2 Participants

**FIGURE 1:**
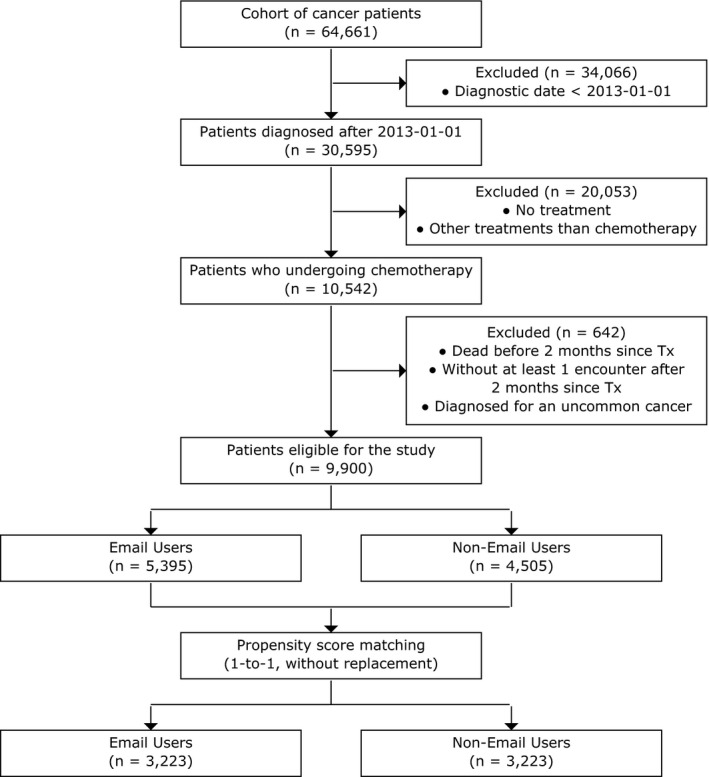
Flowchart of study cohort, 2008–2018

The study included patients diagnosed with cancer who also received chemotherapy treatment at our Cancer Institute between 2013 and 2018. Current Procedural Terminology (CPT) codes and International Classification of Diseases 9 (ICD9) codes were used to identify the first date of chemotherapy treatment in the EHR structured data (Table S1). First‐line and second‐line chemotherapy patients were included in the study. Patients were excluded if they died within 2 months of the start of chemotherapy or if they did not have at least one face‐to‐face visit during the survival period. Finally, we excluded the patients diagnosed with uncommon cancers (i.e., <10 patients per cancer type) to protect patient privacy (Figure 1).

### 2.3 Intervention

Patient generated emails were captured from the MyHealth patient portal. These email correspondences have a structured subject line and the patient must choose from a limited set of choices ("Non‐Urgent Medical Question", "Prescription Question", "Visit Follow‐Up Question", "Test Results Question", "Update My Health Information", "Scheduling Question", "Ordered Test Question"). The body of the email is completely free text and limited to 1000 characters. All emails are triaged to appropriate members—clerical, scheduling, clinical, or other—members of the patients’ healthcare team for action or response.

### 2.4 Study variables

Patients self‐categorized their emails at initiation: “Non‐Urgent Medical Question”, “Patient Medication” (renewal request or question), “Appointment” (schedule, question or cancel), “Test Results”, “Questionnaire” and “Other”. The subject "Other" included undefined subjects (e.g., “visit follow‐up question”). All patient‐initiated emails were included in the study, except for emails with the subject “Questionnaire,” because these emails were not initiated by the patient. Patient‐initiated emails sent regarding to medications or appointments included a comment section where patient concerns could be addressed. Emails that were replies to emails sent from the healthcare team were not considered as they were not patient initiated. Patient demographics and clinical data were captured at the time of diagnosis and first date of treatment, including the following: age at treatment, primary cancer type, gender, insurance payor at treatment, ethnicity, race, and stage at diagnosis. Estimation of household income was calculated based on the US Census data.^9^ Charlson comorbidity score was calculated for each patient based on clinical information available within 2 years before first date of treatment. Deaths were captured from the Stanford Cancer Registry or from the patient health records.

**FIGURE 2:**
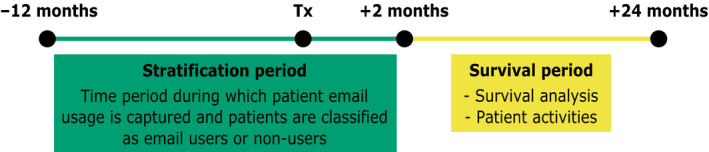
Illustration of the study timeline, including the stratification period and the survival period. Abbreviations: Tx, Treatment. For this study, the stratification period was defined as 12 months prior to 2 months after the first date of chemotherapy treatment (Tx) (green box) and the survival period was defined as 2–24 months after the start of chemotherapy (yellow box). If patients sent at least one email to the healthcare team during the stratification period, they were classified as email users otherwise they were classified as nonemail users, regardless if they sent an email during the survival period. The overall survival rates and patient activities were examined during the survival period

The study design is depicted in Figure 2. For cohort definition, email users were defined as patients who initiated at least one email to their healthcare team (i.e., an inbound email to the healthcare team) in the 12 months prior or 2 months after the start of chemotherapy. Non‐email users were those who did not initiate an email during the stratification period. The stratification period was extended to 2 months after initiation of chemotherapy to maximize the number of email users captured, while also maximizing the survival analysis period (Figure S1). The stratification period was chosen before survival analysis period to avoid the immortal time bias.^10^

### 2.5 Outcome measures

The primary outcome was 2‐year overall survival following the initiation of chemotherapy stratified by email use. Secondary outcomes included the number of patient‐initiated emails, face‐to‐face visits, new and/or refill prescriptions, and telephone calls during the survival period. We also compared the rate of chemotherapy‐related Emergency Department (ED) and In‐Patient (IP) visits during the survival period.^11^ Finally, we examined the healthcare team's response to the patients’ emails. We tabulated as positive responses by the healthcare team to include: a direct reply email from medical staff, appointment scheduling, drug prescription or refill, or phone call from the department who received the email. Healthcare team responses were included if they occurred within 5 days after receiving the patient email.

### 2.6 Statistical analysis

A propensity score‐matched cohort analysis was carried out to reduce bias due to confounding. The propensity score was developed through a multivariable logistic regression model that included the baseline covariates (age, primary cancer type, gender, insurance payor, ethnicity, race, stage, income, Charlson score) and the county of patient residence. A 1‐to‐1 propensity score‐matching was performed, without replacement on the basis of the estimated propensity score of each patient. The match tolerance was set at 0.005 and we used the random method to assign one patient from the email user group to one patient from the non‐email user group.

Using the matched cohort, we performed a bivariate analysis of clinical characteristics between the email users and non‐email users and performed an unpaired t‐test for parametric data and chi‐square/Fisher's exact tests for categorical variables. All statistical tests were two‐sided with a threshold of *p* ≤  0.05 for statistical significance.

Survival curves were generated using Kaplan–Meier analysis. Patients were followed until death or censoring defined by their last face‐to‐face encounter. Patients were stratified by email use and compared using a log‐rank test and Cox proportional hazards regression, adjusting for the baseline covariates.

We compared the patient activities and patient encounters during the survival period, by counting the number of patient‐initiated emails, face‐to‐face visits, new and/or refill prescriptions, and phone calls. To reduce the bias due to the survival rate, we adjusted the number of patient activities and encounters by computing the expected number of observations during the 2‐year survival period.

We compared the rate of chemotherapy‐related ED and IP visits during the survival period by calculating the cumulative incidence functions of the first date of ED or IP visits, with death treated as a competing event. We used the competing risk regression to model risk, without adjustment for baseline covariates. Chemotherapy‐related ED and IP visits were defined according to the Centers for Medicare and Medicaid Services (CMS) endorsed the quality measure, “Admissions and Emergency Department (ED) Visits for Patients Receiving Outpatient Chemotherapy,” which included a primary diagnosis for at least one of the following diagnoses—anemia, dehydration, diarrhea, emesis, fever, nausea, neutropenia, pain, pneumonia, or sepsis.^11^

### 2.7 Bootstrapping & sensitivity analyses

In order to estimate the sampling variability of the email use effect on survival after propensity score matching without replacement, we applied a bootstrapping strategy, as described in a previous study.^12^ The strategy consisted of drawing bootstrap samples from the original (unmatched) dataset, performing propensity score matching and assessing the Cox proportional hazard ratio of email use on survival. The standard deviation of the hazard ratio was used as an estimate of the standard error of hazard ratio of email use with 95% confident intervals. We used a bootstrapping of 100 iterations and a full sample size. To evaluate the sensitivity of the study to missing data, we used the bootstrapping strategy for the full dataset and for a dataset from which we removed patients with unknown values for clinical stage, insurance, and race. Next, we compared the variability between the two datasets.

## 3 RESULTS

### 3.1 Study participants

From a total 64,661 cancer patients identified, 30,595 patients were diagnosed after 1 January 2013, and of these, 10,542 were treated with chemotherapy. After exclusions, we were left with a cohort of 9900 patients, and based on email usage prior to treatment, 5395 patients were classified as email users and 4505 patients as non‐email users (Figure 1).

### 3.2 Propensity score matching

Patient demographics differed significantly between email users and non‐users, with email users having a greater proportion of white, non‐Hispanic privately insured patients with a higher median household income (Table S2). Using propensity score‐matching that included all baseline covariates and the county of residence location, the model matched a group of 3223 email users to a group of 3223 non‐email users. After matching, there were no significant differences in covariate means for both groups (Table 1). The mean age of email users and non‐email users was 59.39 ± 14.69 and 59.41 ± 16.34 years, respectively (*p* = 0.95). The two groups showed similar distributions for gender (*p* = 0.75), ethnicity (*p* = 0.77), race (*p* = 0.72), insurance type (*p* = 0.94), and Charlson co‐morbidity index (*p* = 0.99). The groups were comparable in the distribution of primary cancer histologic types (*p* = 0.99), with a large proportion of breast, lung, or prostate cancer patients for both groups. Estimated mean annual income for email users and non‐email users was $98,350.93 ± $37,361.98 and $98,266.71 ± $36,961.44, respectively (*p* = 0.93). Stage at diagnosis was missing for approximately 30% of patients in both groups.

**TABLE 1:** Patient characteristics after propensity score matching, stratified by email use*

Variable		Email users	Nonemail users
Total, No. (%)		3223 (50.00)	3223 (50.00)
Age at treatment (years), Mean ±SD		59.39 ± 14.69	59.41 ± 16.34
Gender, No. (%)	Female	1,554 (49.78)	1,568 (50.22)
	Male	1,669 (50.21)	1,655 (49.79)
Insurance payor at treatment, No. (%)	Private	1,240 (50.24)	1,228 (49.76)
	Medicare	1,214 (49.77)	1,225 (50.23)
	Medicaid	291 (49.07)	302 (50.93)
	Unknown	478 (50.53)	468 (49.47)
Ethnicity, No. (%)	Non‐Hispanic/Non‐Latino	2,810 (49.92)	2,819 (50.08)
	Hispanic/Latino	413 (50.55)	404 (49.45)
Race, No. (%)	White	1,804 (49.68)	1,827 (50.32)
	Asian	661 (49.62)	671 (50.38)
	Black	92 (48.68)	97 (51.32)
	Other^a^	39 (54.93)	32 (45.07)
	Unknown	627 (51.27)	596 (48.73)
Stage at diagnosis, No. (%)	0	18 (43.90)	23 (56.10)
	1	400 (49.32)	411 (50.68)
	2	551 (49.91)	553 (50.09)
	3	406 (50.37)	400 (49.63)
	4	841 (50.30)	831 (49.70)
	Unknown	1,007 (50.05)	1,005 (49.95)
Household Annual Income Estimation based on US Census data^b^, Mean ±SD		$98,350.93 ± $37,361.98	$98,266.71 ± $36,961.44
Charlson Score^b^, Mean ±SD		5.17 ± 3.32	5.17 ± 3.45
Primary cancer, No. (%)	Breast	392 (49.75)	396 (50.25)
	Lung	295 (51.13)	282 (48.87)
	Pancreatic & biliary	401 (50.70)	390 (49.30)
	Blood, bone marrow, & hematopoietic system	465 (50.32)	459 (49.68)
	Lymphoma	394 (49.68)	399 (50.32)
	Head & neck	222 (51.27)	211 (48.73)
	Colorectal	193 (50.79)	187 (49.21)
	Prostate	144 (50.70)	140 (49.30)
	Upper gastrointestinal tract	117 (47.76)	128 (52.24)
	Ovarian	85 (45.45)	102 (54.55)
	Cervical & uterine	101 (48.79)	106 (51.21)
	Bladder	77 (50.33)	76 (49.67)
	Kidney & ureter	47 (48.45)	50 (51.55)
	Skin: melanoma	71 (48.63)	75 (51.37)
	Brain & other nervous system	73 (51.41)	69 (48.59)
	Connective & soft tissue	49 (50.00)	49 (50.00)
	Testicular	26 (47.27)	29 (52.73)
	Retroperitoneum & peritoneum	23 (48.94)	24 (51.06)
	Bone & joints	17 (43.59)	22 (56.41)
	Pleura	7 (58.33)	5 (41.67)
	Meninges	2 (50.00)	2 (50.00)
	Mediastinum	7 (50.00)	7 (50.00)
	Spleen	6 (50.00)	6 (50.00)
	Vagina & labia	4 (57.14)	3 (42.86)
	Orbit & lacrimal gland	4 (50.00)	4 (50.00)
	Vulva	1 (33.33)	2 (66.67)

^a^ Other race category includes Pacific Islander and Native American.

^b^ Continuous variable per point.

* All *p*‐values were >0.7.

### 3.3 Survival analysis

**FIGURE 3:**
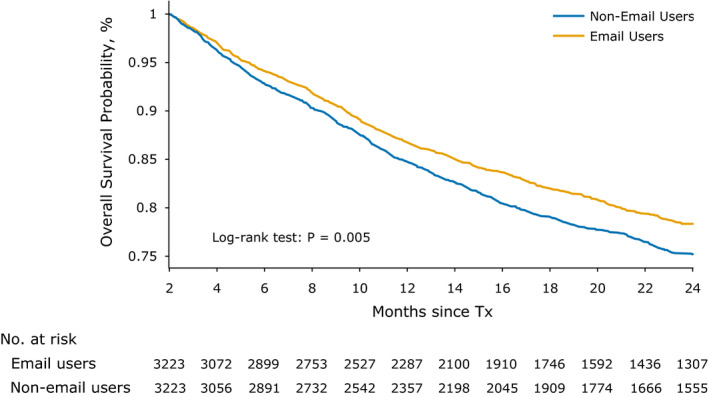
Overall 2‐year survival among cancer chemotherapy patients, stratified by email users versus nonemail users. Abbreviations: Tx, Treatment. The median 2‐year survival was 78.29% (95% CI, 79.85%‐76.63%) for email users and 75.22% (95% CI, 76.79%‐73.55%) for nonemail users (difference, 5 months; *p* < 0.01). Email users were defined as patients who sent at least one email 12 months before through 2 months after the first date of chemotherapy treatment

Overall survival was calculated between 2 months after the first date of chemotherapy treatment and 24 months. The median follow‐up time was 21 months (interquartile range, 11.1‐37.5). The median 2‐year survival was 78.29% (95% CI, 79.85%‐76.63%) for email users and 75.22% (95% CI, 76.79%‐73.55%) for non‐email users (difference, 5 months; *p* < 0.01) (Figure 3). A Cox proportional hazards model, adjusting for the baseline covariates, showed that email users had a significant survival benefit, with a hazard ratio of 0.80 (95 CI 0.72‐0.90; *p* < 0.001) (Table S3).

We used a bootstrapping strategy to estimate the sampling variability of the survival benefit to email use for the full dataset and for the dataset with no unknown values (i.e., a dataset in which patients with unknown clinical stage, insurance or race were excluded). For both datasets, the average hazard ratio for email use was significant. The full dataset achieved an average hazard ratio of 0.776 ± 0.059 (95% CI 0.68‐0.90) and the dataset with no unknown values reached an average hazard ratio of 0.716 ± 0.085 (95% CI 0.57‐0.87).

### 3.4 Patient activities during survival period

Frequency of interactions with the healthcare system of the 6276 email users and non‐users during the survival period is summarized in Table 2. In the non‐email user group, there were some patients who sent an email during the survival period, although they did not send an email during the stratification period (Figure 2). During that same period, email users were more likely to have more face‐to‐face visits, drug prescriptions and phone calls and were less likely to miss appointments compared to non‐users (*p* < 0.01 for all of these). There were significantly fewer chemotherapy related inpatient admissions for email users (10.7% of email users vs. 12.7% of non‐users, *p* = 0.015) and fewer, although not significant, ED‐visits (5.9% for email users vs. 6.6% for non‐users, *p* = 0.25) using criteria defined by the CMS quality measure (Figure S2A and B).

**TABLE 2:** Estimation of patient activities during the survival period, stratified by email and nonemail users^a^

Activity	Email users	Nonemail users	*p*
Initiated emails, Mean ±SD	25.93 ± 35.01	3.51 ± 10.62	<0.001
Face‐to‐face visits, Mean ±SD	62.95 ± 56.74	50.14 ± 51.65	<0.001
Missed Face‐to‐face visits, Mean ±SD	0.41 ± 1.21	0.5 ± 1.46	0.005
Drug prescriptions, Mean ±SD	27.73 ± 35.92	21.19 ± 31.35	<0.001
Phone calls, Mean ±SD	18.16 ± 20.88	16.13 ± 21.37	<0.001

^a^ The adjusted number of observations for each patient is the expected number of observations until the end of 24 months after treatment.

### 3.5 Healthcare provider responses to patient emails

**FIGURE 4:**
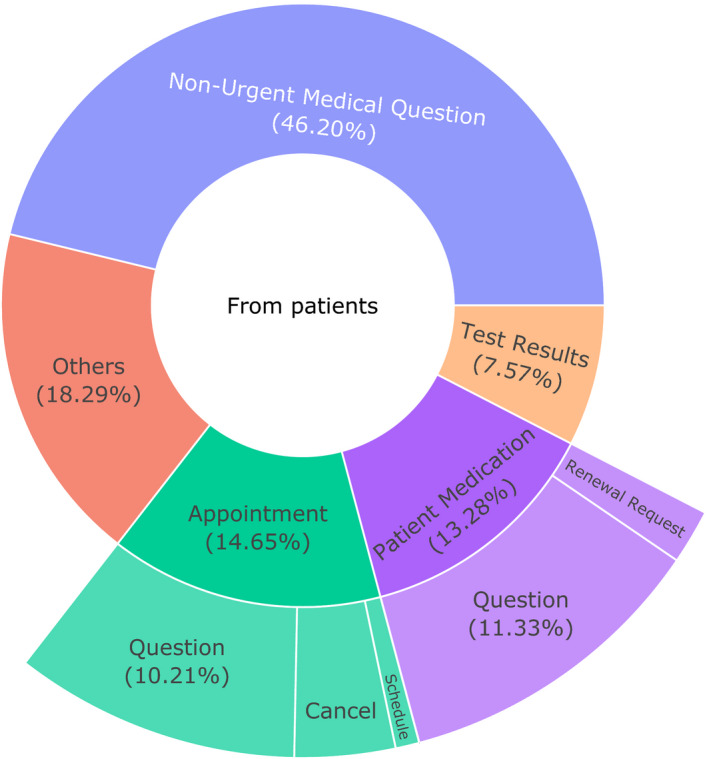
Sunburst chart of the patient‐initiated emails’ subjects during the survival period. Patient‐initiated emails were categorized as: “Non‐Urgent Medical Question,” “Patient Medication” (renewal request or question), “Appointment” (schedule, question or cancel), “Test Results,” and “Other.” The subject “Other” included undefined subjects (e.g., “visit follow‐up question”). In total, 72,003 patient‐initiated emails were initiated by the 6,446 patients during the survival period (from 2 months to 24 months after the start of chemotherapy). Patient emails that were a reply to an email sent from the healthcare team were not considered, as they were not patient‐initiated

In total, 72,003 patient‐generated emails were initiated by the 6446 patients during the survival period (Figure 4). Patient generated emails could be categorized based on subject line, including: Non‐Urgent Medical Question (33,268 emails, 46.2%); Appointment (14.7%); and Patient Medication (13.3%). Only a small fraction of the emails characterized as “Appointment” requested a new appointment (5.8% of all “Appointment” emails), likely because treatment protocols follow discrete appointment templates that were scheduled in advance by the medical team. The remaining 13,166 emails (18.3%) could not be classified by subject, because they included diverse personalized email subjects.

**FIGURE 5:**
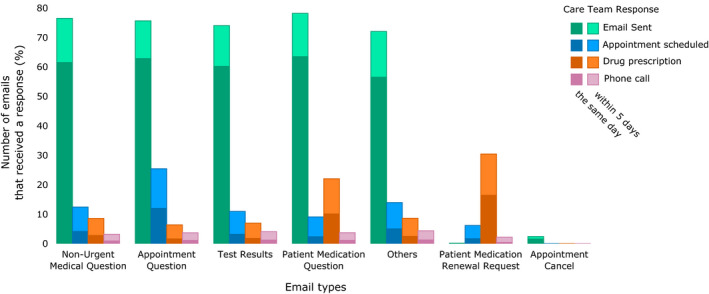
Percentage of emails that receive a response from the healthcare team, stratified by email subject and response type. Patient‐initiated emails were categorized as follows: “Non‐Urgent Medical Question,” “Patient Medication” (renewal request or question), “Appointment” (schedule, question or cancel), “Test Results,” and “Other.” The subject “Other” included undefined subjects (e.g., “visit follow‐up question”). In total, 72,003 patient‐initiated emails were initiated by the 6446 patients during the survival period (from 2 months to 24 months after the start of chemotherapy). Patient emails that were a reply to an email sent from the healthcare team were not considered, as they were not patient‐initiated. Positive responses to patient‐initiated emails by the healthcare team include the following: a direct reply email from medical staff, appointment scheduling, drug prescription or refill, or phone call from the department who received the email. Healthcare team responses were included if they occurred within 5 days after receiving the patient email

The analysis of the healthcare team's response to a patient email is presented in Figure 5. For emails with the subject lines “Non‐Urgent Medical Question,” “Appointment Question,” “Medication Question,” and “Test Results,” the healthcare team responded by email within 1 day for 61.8% of messages, and within 2 days for 73.2% (Figure 5). For emails with the subject line "Medication Renewal Request," a drug prescription was ordered within 5 days for 33.7% of emails after receiving the request. Finally, only 3.5% of emails was followed by a phone call from the healthcare team after 5 days (Table S4).

## 4 DISCUSSION

For cancer patients receiving chemotherapy for diverse malignancies, email usage was associated with significantly improved overall survival within 24 months of chemotherapy initiation compared to non‐email use. These results suggest that email use might represent a proxy for greater engagement with the healthcare system. Our findings suggest that email communication may be associated with patient health outcomes. Given the wide availability, usage and comfort with email as a means of communication in non‐healthcare contexts, encouraging patient email use could be an important means of improving healthcare outcomes, as well as a novel data source for understanding patients’ symptoms and general well‐being.

Our finding of improved survival among patients who were frequent email users is consistent with a recent prospective randomized trial showing that structured communications between the healthcare team and cancer patients was associated with improved overall survival.^13^, ^14^ In the clinical trial of Basch et al, patients with regularly scheduled, scripted communication with the healthcare team had a survival benefit after enrollment at 7 years of follow‐up. Intriguingly, we found a 5‐month survival benefit for patients engaging in ad hoc, unstructured email communication as part of their standard care.

To date, most health‐related information that is patient‐generated has been captured prospectively from validated instruments (e.g., structured surveys).^13^, ^15^, ^16^ Indeed, this information is easier to compute and analyze and has greatly advanced our understanding of patients’ values and symptoms. Yet, these studies often have selection bias, limited sample sizes, and may not be generalizable to the average patient in routine care. Whether unstructured email communication through a secure portal in the EHR can provide similar survival benefits compared to provider‐initiated structured program, as in Basch et al.^14^, is worth further testing. However, unstructured email communications provide opportunities for each patient to drive the level of communication they need, on topics they define, and when convenient to them, rather than forcing them to only address issues defined by the healthcare team (e.g., survey instruments) at inconvenient or irrelevant times.

The patients’ voice is often missing from routine clinical care.^17^ The ability to leverage the patients’ voice through emails could present a paradigm shift in the way patients engage in and report their cancer experience. Recently, the CMS proposed a new payment model for oncology that requires the monitoring of patient‐reported outcomes during treatment, Oncology Care First.^11^ However, there are concerns about the ability of health systems and patients to engage in PRO reporting systems.^18^ Developing informatics capacities that could systematically monitor patient‐generated emails for the treatment symptoms and side‐effects is an easy first step. Our innovative approach to capture of PROs could help providers and practices meet the Oncology Care First model requirements and improve outcomes.

Similar to other email studies,^1^ we found the majority of patient‐initiated emails had a timely response by the care team, which included a prescription, appointment, phone call, or email response. This suggests that the emails sent by the patient identified actionable concerns and/or legitimate healthcare needs. Patient‐initiated emails support good patient–physician communication, which improves patient outcomes and quality of life, particularly for patients with serious, life‐limiting diseases such as advanced cancer.^19^, ^20^ In addition, patients sending emails to their providers were high utilizers of the healthcare system evidenced by more frequent telephone calls, face‐to‐face visits, and prescriptions—even after controlling for severity of illness. However, these patients also had fewer chemotherapy‐related hospital admissions. Indeed, patients engaging with the healthcare team likely have early detection of adverse treatment‐related side effects, and more timely responses, which could explain the benefits found in this study.

As the adoption of patient portals increases, biomedical informatics techniques will be needed to assist in understanding and managing the growing volumes of email communication. Artificial Intelligence technologies to mine this trove of emails, such as Natural Language Processing and Machine Learning, will be necessary to triage important messages to healthcare teams in a timely fashion. In addition, these methods could support a new data stream of patient‐centered outcomes that could be used to understand and improve the healthcare experience. Commercial products using artificial intelligence to capture patient's symptoms and health concerns from emails to guide scheduling, and online tools that use chatbots to triage patient requests for appointments are already under development.^21^, ^22^

The interpretation of this study's results has limitations. First, we used email as a proxy for patient engagement, which might not be an appropriate assumption. However, patients in the email user group also had more telephone calls and face‐to‐face visits, indicating a high level of engagement. Furthermore, patients who were frequent email users sent seven times more emails during the survival period, thereby validating the assumption that pretreatment email use identifies a high use cohort during treatment. Second, email usage is only one form of communication and 45% of our population did not initiate an email prior to their chemotherapy treatment. Future analytic work should expand to include other forms of communication, such as telephone calls and text messages, and clinical trials of interventions should test interventions to expand email use. Third, our data are derived from a single healthcare system and may not be generalizable to other settings. Despite these shortcomings, patient email communications have promise to improve outcomes, communication, and engagement, and could be used to improve the quality of care and patient experience. Fourth, we were limited to capture hospitalizations and ED visits or deaths that occurred outside our setting. However, providers often follow their patients and indicate death in the EHR after follow‐up—and this information we captured. Finally, we are aware that this study may suffer from possible residual unmeasured confounders even after propensity score matching, such as patient education information, or missing data, as the stage was missing in 30% of patients. However, the bootstrapping showed a high robustness in our findings, by estimating the sampling variability of the model, with and without unknown data.

## 5 CONCLUSIONS

In a real‐world cohort of cancer patients receiving chemotherapy, patients initiating email conversations with their care team had better 2‐year survival rates compared to patients not emailing their providers prior to treatment. Patients who were frequent email users showed higher levels of engagement and had fewer chemotherapy‐related inpatient admissions and ED visits during the survival period. In the future, AI methods could be used to analyze email content to triage messages, capture the patient voice, and develop automated systems to address patient needs and to potentially reduce healthcare provider burnout. Patient‐generated emails provide a new path for patients to be active participants in their care, which may improve patient–clinician communication, patient experience, and overall outcomes of care.

## CONFLICT OF INTEREST

The authors declare that they have no known competing financial interests or personal relationships that could have appeared to influence the work reported in this paper.

## AUTHORS’ CONTRIBUTIONS

Dr. Hernandez‐Boussard takes responsibility for the integrity of the data and the accuracy of the data analysis. Concept and design: All authors. Statistical analysis: Jean Coquet and Tina Hernandez‐Boussard. Collection of data: Jean Coquet. Interpretation of data: All authors. Drafting of the manuscript: Jean Coquet. Critical revision of the manuscript: All authors. Administrative, technical, or material support: Tina Hernandez‐Boussard. Study supervision: Tina Hernandez‐Boussard.

## Supporting information

Fig S1

Fig S2

Tables S1‐S4

## Data Availability

Data sharing is not applicable to this article as the data that has been used is confidential.
