# Supplementary figures and images for: Association between patient‐initiated emails and overall 2‐year survival in cancer patients undergoing chemotherapy: Evidence from the real‐world setting
Source: Cancer Med. 2020 Sep 28;9(22):8552–61. doi: 10.1002/cam4.3483 (PMC7666724; doi:10.1002/cam4.3483)

Number of email users

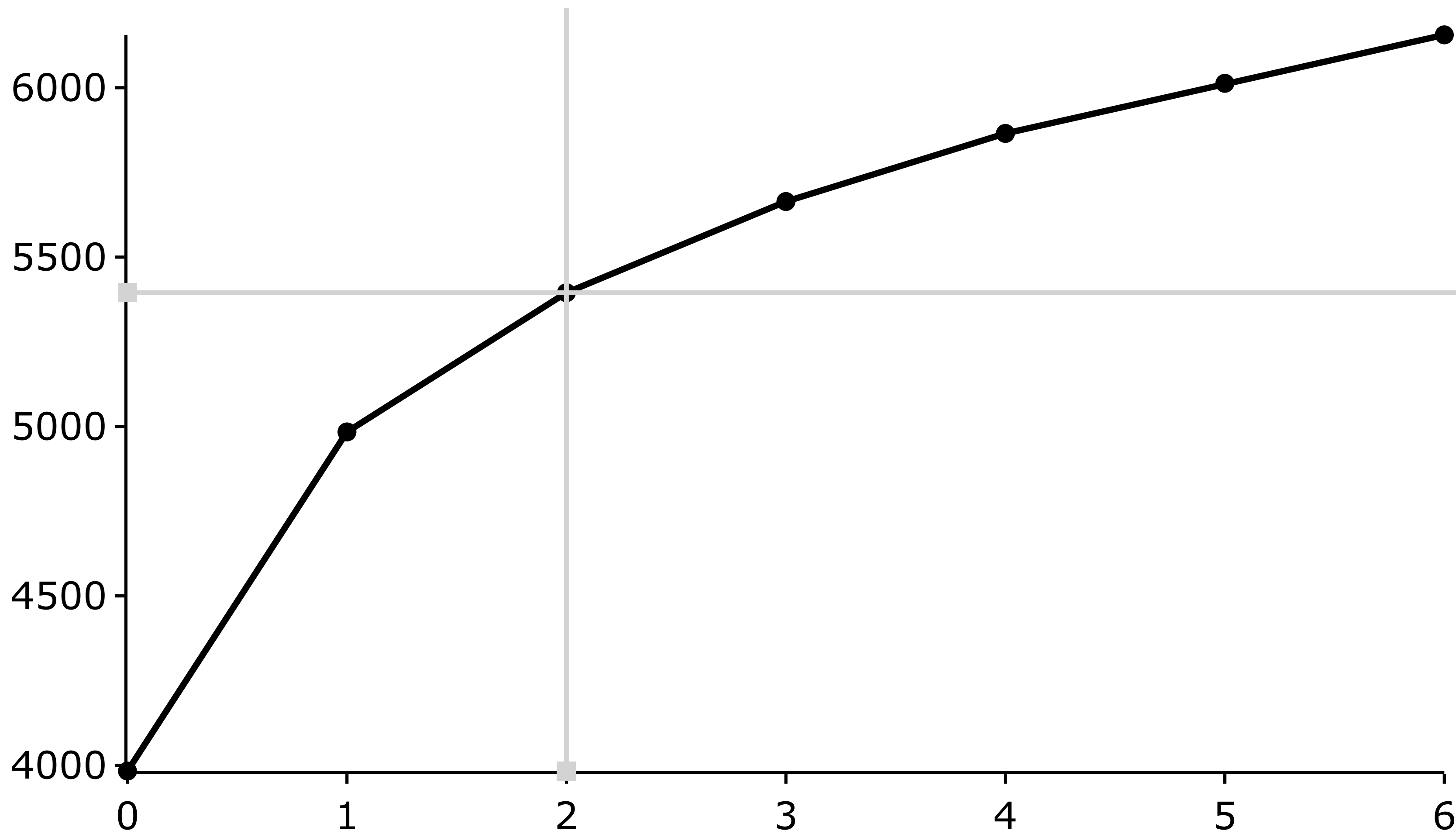

Months after Tx

Supplement: Supplementary file 1 — Fig S1 [file CAM4-9-8552-s001.pdf]

(A)

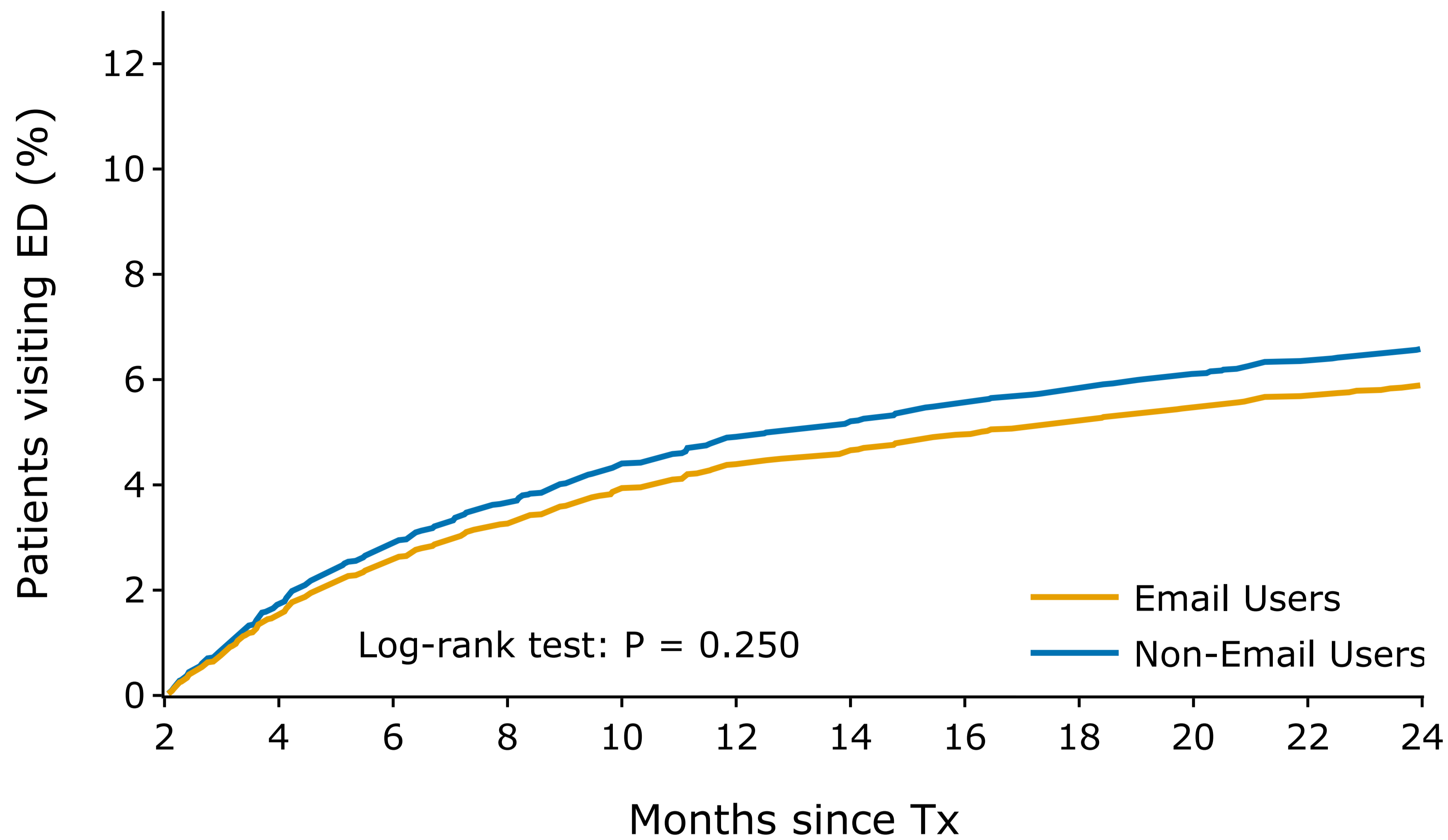

(B)

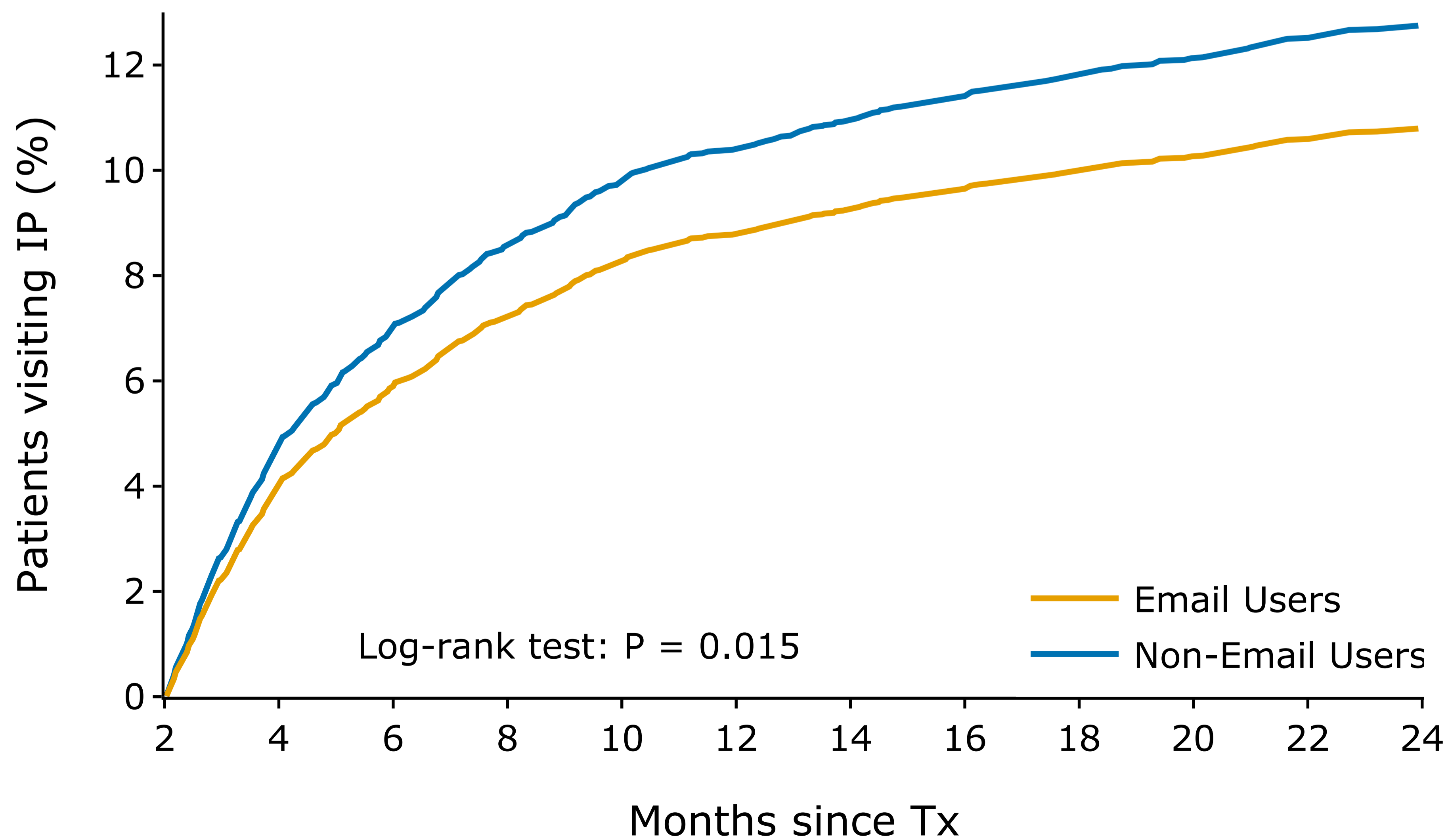

Supplement: Supplementary file 2 — Fig S2 [file CAM4-9-8552-s002.pdf]
