# Supplementary material for: Association between patient‐initiated emails and overall 2‐year survival in cancer patients undergoing chemotherapy: Evidence from the real‐world setting
Source: Cancer Med. 2020 Sep 28;9(22):8552–61. doi: 10.1002/cam4.3483 (PMC7666724; doi:10.1002/cam4.3483)
Supplement: Supplementary file 3 — Tables S1‐S4 [file CAM4-9-8552-s003.docx]

# ONLINE-ONLY SUPPLEMENTS

**Table S1. CPT and ICD-9 codes used to identify chemotherapy treatment.**

**Table S2. Demographic data of cancer chemotherapy patients before propensity score matching, stratified by email users and non-email users.**

**Table S3. Cox proportional hazards model for overall 2-year survival among cancer chemotherapy patients.**

**Table S4. Healthcare team response to patient-initiated emails during the survival period, stratified by email subject.**

**Figure S1. Cumulative number of email users in function of stratification period.**

**Figure S2. Competing Risks Regression Models of Chemotherapy-related Emergency Department (A) and Inpatient (B) visit rate during the survival study period.**

**Table S1. CPT and ICD-9 codes used to identify chemotherapy treatment.**

| Ontology | Codes |
| --- | --- |
| ICD9 | 99.25 |
| CPT | 96445, 96406, 96405, 96411, 96409, 96415, 96417, 96416, 96413, 96402, 96401, 96423, 96425, 96422, 96420, 96440, 96446, 96450, 96542, 96549 |

Abbreviations: CPT, Current Procedural Terminology; ICD-9, International Classification of Diseases 9.

**Table S2. Demographic data of cancer chemotherapy patients before propensity score matching, stratified by email users and non-email users.***

| Variable |  | Email Users | Non-Email Users |
| --- | --- | --- | --- |
| Total, No. (%) | | 5,395 (54.49) | 4,505 (45.51) |
| Age at Treatment (years), Mean ± SD | | 59.04 ± 14.32 | 58.44 ± 16.82 |
| Gender, No. (%) | Female | 2,755 (56.51) | 2,120 (43.49) |
|  | Male | 2,640 (52.54) | 2,385 (47.46) |
| Insurance Payor at Treatment, No. (%) | Private | 2,382 (59.85) | 1,598 (40.15) |
|  | Medicare | 1,869 (52.19) | 1,712 (47.81) |
|  | Medicaid | 350 (38.50) | 559 (61.50) |
|  | Unknown | 794 (55.52) | 636 (44.48) |
| Ethnicity, No. (%) | Non-Hispanic/Non-Latino | 4,946 (58.27) | 3,542 (41.73) |
|  | Hispanic/Latino | 449 (31.80) | 963 (68.20) |
| Race, No. (%) | White | 3,195 (58.66) | 2,252 (41.34) |
|  | Asian | 1,277 (62.14) | 778 (37.86) |
|  | Black | 104 (35.86) | 186 (64.14) |
|  | Other^†^ | 50 (45.45) | 60 (54.55) |
|  | Unknown | 769 (38.49) | 1,229 (61.51) |
| Stage at Diagnosis, No. (%) | 0 | 45 (65.22) | 24 (34.78) |
|  | 1 | 728 (56.17) | 568 (43.83) |
|  | 2 | 1,023 (58.69) | 720 (41.31) |
|  | 3 | 666 (54.28) | 561 (45.72) |
|  | 4 | 1,377 (55.59) | 1,100 (44.41) |
|  | Unknown | 1,556 (50.39) | 1,532 (49.61) |
| Household Annual Income Estimation based on US Census data^‡^, Mean ± SD | | $10,4152.38 ± $39,501.71 | $91,774.56 ± $36,075.63 |
| Charlson Score^‡^, Mean ± SD | | 5.52 ± 3.37 | 4.87 ± 3.35 |
| Primary cancer, No. (%) | Breast | 939 (68.04) | 441 (31.96) |
|  | Lung | 522 (61.70) | 324 (38.30) |
|  | Pancreatic & Biliary | 524 (46.13) | 612 (53.87) |
|  | Blood, Bone Marrow, & Hematopoietic System | 515 (37.48) | 859 (62.52) |
|  | Lymphoma | 448 (40.69) | 653 (59.31) |
|  | Head & Neck | 450 (64.38) | 249 (35.62) |
|  | Colorectal | 369 (62.86) | 218 (37.14) |
|  | Prostate | 370 (69.94) | 159 (30.06) |
|  | Upper Gastrointestinal Tract | 209 (58.71) | 147 (41.29) |
|  | Ovarian | 176 (58.67) | 124 (41.33) |
|  | Cervical & Uterine | 146 (48.18) | 157 (51.82) |
|  | Bladder | 140 (63.35) | 81 (36.65) |
|  | Kidney & Ureter | 125 (69.06) | 56 (30.94) |
|  | Skin: Melanoma | 112 (53.59) | 97 (46.41) |
|  | Brain & Other Nervous System | 108 (53.20) | 95 (46.80) |
|  | Connective & Soft Tissue | 77 (53.85) | 66 (46.15) |
|  | Testicular | 56 (59.57) | 38 (40.43) |
|  | Retroperitoneum & Peritoneum | 33 (54.10) | 28 (45.90) |
|  | Bones & Joints | 20 (30.30) | 46 (69.70) |
|  | Pleura | 14 (58.33) | 10 (41.67) |
|  | ﻿Meninges | 10 (71.43) | 4 (28.57) |
|  | Mediastinum | 9 (45.00) | 11 (55.00) |
|  | Spleen | 8 (57.14) | 6 (42.86) |
|  | Vagina & Labia | 7 (50.00) | 7 (50.00) |
|  | Orbit & Lacrimal Gland | 6 (46.15) | 7 (53.85) |
|  | Vulva | 2 (16.67) | 10 (83.33) |

* All p-values were <0.001 except Age which had a p-value of 0.056

^†^ Other race category includes Pacific Islander and Native American.

^‡^ Continuous variable per point.

**Table S3. Cox proportional hazards model for overall 2-year survival among cancer chemotherapy patients.**

| ﻿Variable |  | HR | 95% CI | p |
| --- | --- | --- | --- | --- |
| Email User | | 0.802 | 0.718 - 0.896 | <0.001 |
| Age at Treatment (years) | | 1.016 | 1.011 - 1.021 | <0.001 |
| Gender | Male |  | | |
|  | Female | 0.931 | 0.825 - 1.051 | 0.249 |
| Insurance Payor at Treatment | Private |  | | |
|  | Medicare | 0.854 | 0.734 - 0.993 | 0.041 |
|  | Medicaid | 1.219 | 0.995 - 1.492 | 0.055 |
|  | Unknown | 0.593 | 0.486 - 0.722 | <0.001 |
| Ethnicity | Non-Hispanic/Non-Latino |  | | |
|  | Hispanic/Latino | 0.972 | 0.779 - 1.213 | 0.800 |
| Race | White |  | | |
|  | Asian | 1.069 | 0.926 - 1.233 | 0.362 |
|  | Black | 1.233 | 0.893 - 1.703 | 0.203 |
|  | Other^†^ | 1.266 | 0.743 - 2.157 | 0.386 |
|  | Unknown | 1.177 | 0.979 - 1.414 | 0.083 |
| Stage at Diagnosis | 0 |  | | |
|  | 1 | 3.966 | 0.544 - 28.906 | 0.174 |
|  | 2 | 4.840 | 0.667 - 35.126 | 0.119 |
|  | 3 | 6.476 | 0.890 - 47.132 | 0.065 |
|  | 4 | 11.919 | 1.646 - 86.285 | 0.014 |
|  | Unknown | 7.235 | 0.997 - 52.533 | 0.050 |
| Household Annual Income Estimation based on US Census data^§^ | | 1.000 | 1.000 - 1.000 | 0.689 |
| Charlson Score^§^ | | 1.075 | 1.056 - 1.096 | <0.001 |
| Primary cancer | Blood, Bone Marrow, And Hematopoietic System |  | | |
|  | Lung | 1.047 | 0.809 - 1.355 | 0.725 |
|  | Pancreatic & Biliary | 1.141 | 0.892 - 1.460 | 0.295 |
|  | Breast | 0.289 | 0.203 - 0.411 | <0.001 |
|  | Lymphoma | 0.497 | 0.375 - 0.657 | <0.001 |
|  | Head & Neck | 0.372 | 0.270 - 0.512 | <0.001 |
|  | Colorectal | 0.350 | 0.246 - 0.498 | <0.001 |
|  | Prostate | 0.219 | 0.135 - 0.355 | <0.001 |
|  | Upper Gastrointestinal Tract | 1.215 | 0.908 - 1.625 | 0.190 |
|  | Ovarian | 0.375 | 0.238 - 0.592 | <0.001 |
|  | Cervical & Uterine | 0.841 | 0.585 - 1.210 | 0.351 |
|  | Bladder | 0.703 | 0.451 - 1.096 | 0.120 |
|  | Kidney & Ureter | 0.775 | 0.483 - 1.244 | 0.291 |
|  | Skin: Melanoma | 0.713 | 0.467 - 1.088 | 0.116 |
|  | Brain & Other Nervous System | 3.213 | 2.448 - 4.217 | <0.001 |
|  | Connective & Soft Tissue | 1.084 | 0.688 - 1.707 | 0.729 |
|  | Testicular | 0.587 | 0.255 - 1.350 | 0.210 |
|  | Retroperitoneum & Peritoneum | 1.186 | 0.687 - 2.049 | 0.541 |
|  | Bones & Joints | 0.241 | 0.059 - 0.980 | 0.047 |
|  | Pleura | 0.733 | 0.231 - 2.322 | 0.597 |
|  | ﻿Other^‡^ | 0.904 | 0.474 - 1.721 | 0.758 |

^†^ Other race category includes Pacific Islander and Native American

^‡^ Other primary cancer category includes ﻿Meninges, Mediastinum, Spleen, Vagina & Labia, Orbit & Lacrimal, Gland and Vulva.

^§^ Continuous variable per point.

**Table S4. Healthcare team response to patient-initiated emails during the survival period, stratified by email subject.**

| Care Team Response | Days | Appointment | | Non-Urgent Medical Question | Patient Medication | | Other | Test Results |
| --- | --- | --- | --- | --- | --- | --- | --- | --- |
|  |  | Cancel | Question |  | Question | Renewal Request |  |  |
| Email Sent, % | 0 | 1.6 | 62.8 | 61.5 | 63.5 | 0.1 | 56.5 | 60.2 |
|  | 1 | 2.0 | 68.7 | 69.1 | 71.2 | 0.1 | 64.5 | 66.5 |
|  | 2 | 2.2 | 72.8 | 73.1 | 75.5 | 0.1 | 68.7 | 70.3 |
|  | 3 | 2.3 | 74.3 | 75.0 | 77.3 | 0.1 | 70.6 | 72.4 |
|  | 4 | 2.4 | 75.1 | 76.0 | 77.9 | 0.1 | 71.6 | 73.5 |
|  | 5 | 2.4 | 75.6 | 76.5 | 78.2 | 0.1 | 72.0 | 74.0 |
| Appointment scheduled, % | 0 | 0.0 | 16.0 | 5.5 | 3.2 | 2.1 | 6.6 | 4.3 |
|  | 1 | 0.0 | 21.8 | 8.2 | 5.3 | 3.1 | 10.0 | 7.1 |
|  | 2 | 0.0 | 25.5 | 10.5 | 6.8 | 4.1 | 12.5 | 9.2 |
|  | 3 | 0.0 | 28.3 | 12.5 | 8.6 | 5.5 | 14.7 | 11.2 |
|  | 4 | 0.0 | 30.7 | 14.2 | 10.1 | 7.0 | 16.5 | 12.6 |
|  | 5 | 0.0 | 32.8 | 16.1 | 12.0 | 8.2 | 18.1 | 14.5 |
| Drug prescription, % | 0 | 0.0 | 3.9 | 4.9 | 12.2 | 17.9 | 4.9 | 4.7 |
|  | 1 | 0.0 | 6.9 | 7.5 | 17.1 | 23.8 | 7.6 | 7.7 |
|  | 2 | 0.0 | 9.3 | 10.0 | 21.2 | 27.5 | 9.9 | 9.8 |
|  | 3 | 0.0 | 11.6 | 12.1 | 24.2 | 30.2 | 11.9 | 11.8 |
|  | 4 | 0.0 | 13.3 | 14.1 | 26.4 | 31.9 | 13.7 | 13.6 |
|  | 5 | 0.0 | 15.3 | 16.0 | 28.6 | 33.7 | 15.8 | 16.0 |
| Phone call, % | 0 | 0.0 | 1.1 | 0.9 | 1.1 | 0.4 | 1.3 | 1.2 |
|  | 1 | 0.0 | 1.8 | 1.5 | 1.9 | 1.2 | 2.1 | 1.9 |
|  | 2 | 0.0 | 2.4 | 2.0 | 2.4 | 1.4 | 2.8 | 2.4 |
|  | 3 | 0.0 | 2.9 | 2.5 | 2.9 | 1.8 | 3.3 | 3.0 |
|  | 4 | 0.0 | 3.3 | 2.8 | 3.3 | 1.9 | 3.8 | 3.4 |
|  | 5 | 0.0 | 3.7 | 3.2 | 3.7 | 2.2 | 4.4 | 4.1 |
| Total emails, No. | | 2,588 | 7,350 | 33,268 | 8,158 | 1,406 | 13,166 | 5,454 |

**Figure S1. Cumulative number of email users in function of stratification period.**


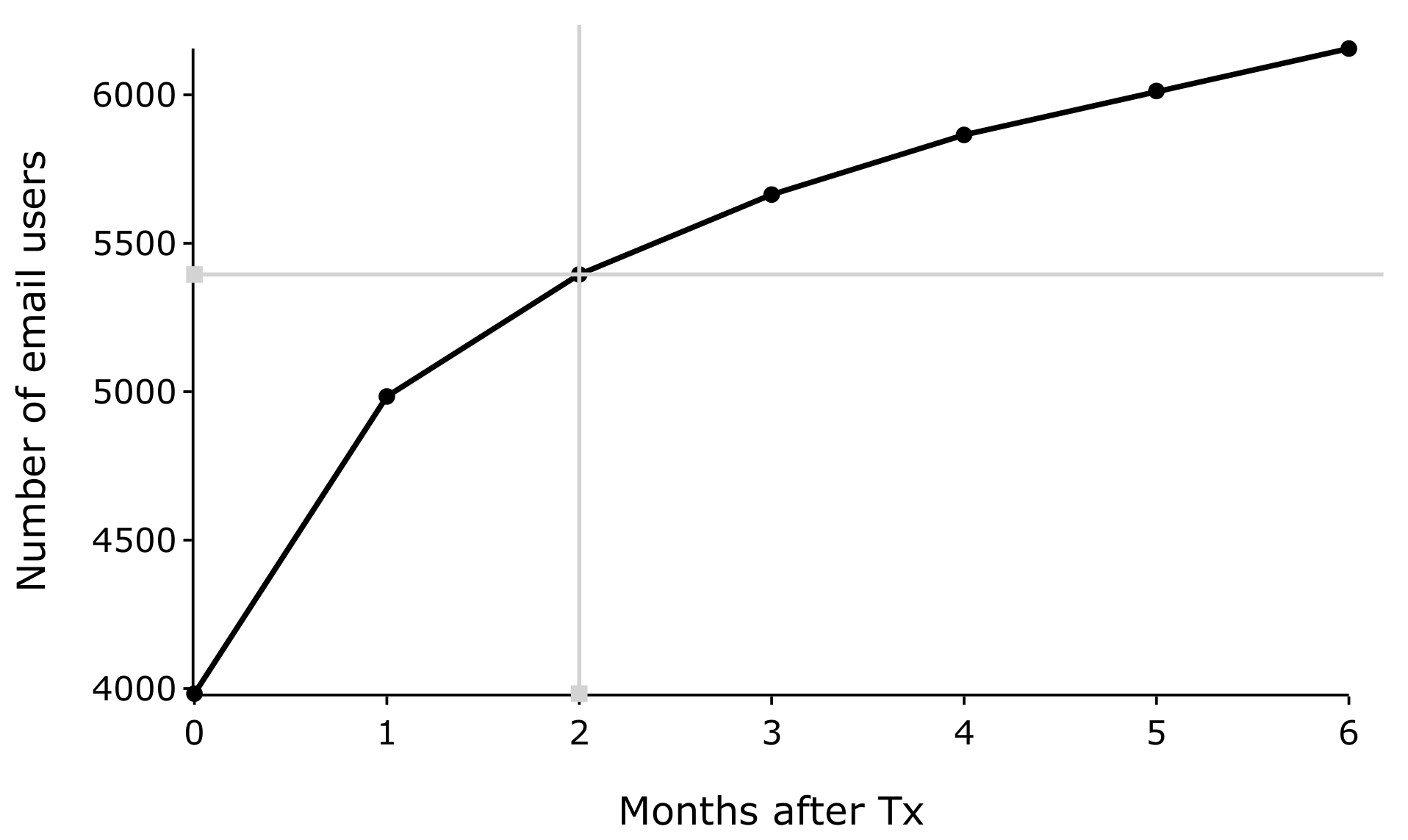


Abbreviations: Tx, Treatment.

**Figure S2. Competing Risks Regression Models of Chemotherapy-related Emergency Department (A) and Inpatient (B) visit rate during the survival study period.**


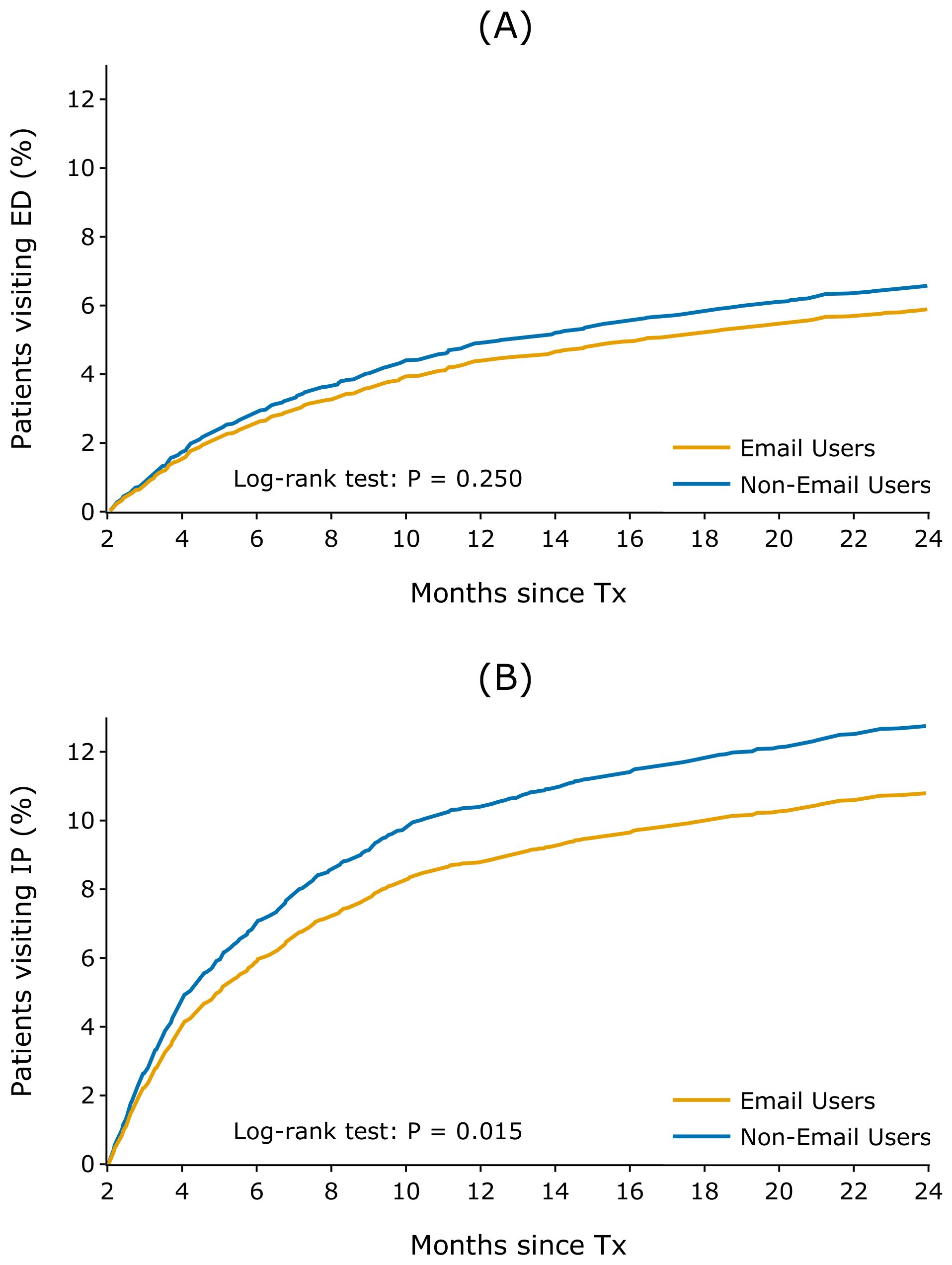


Abbreviations: Tx, Treatment; ED: Emergency Department; IP: Inpatient admission.
